# Supplementary material for: Conway–Bromage–Lyndon (CBL): an exact, dynamic representation of k-mer sets
Source: Bioinformatics. 2024 Jun 28;40(Suppl 1):i48–57. doi: 10.1093/bioinformatics/btae217 (PMC11211824; doi:10.1093/bioinformatics/btae217)
Supplement: btae217_Supplementary_Data [file btae217_supplementary_data.zip › btae217_Supplementary_Data/Martayan.215.sup.pdf]

Appendix

Supplementary results

Parametrization of CBL

Using similar bacterial datasets to those described in the main document, we analyze the impact of the prefix size in CBL's performance.

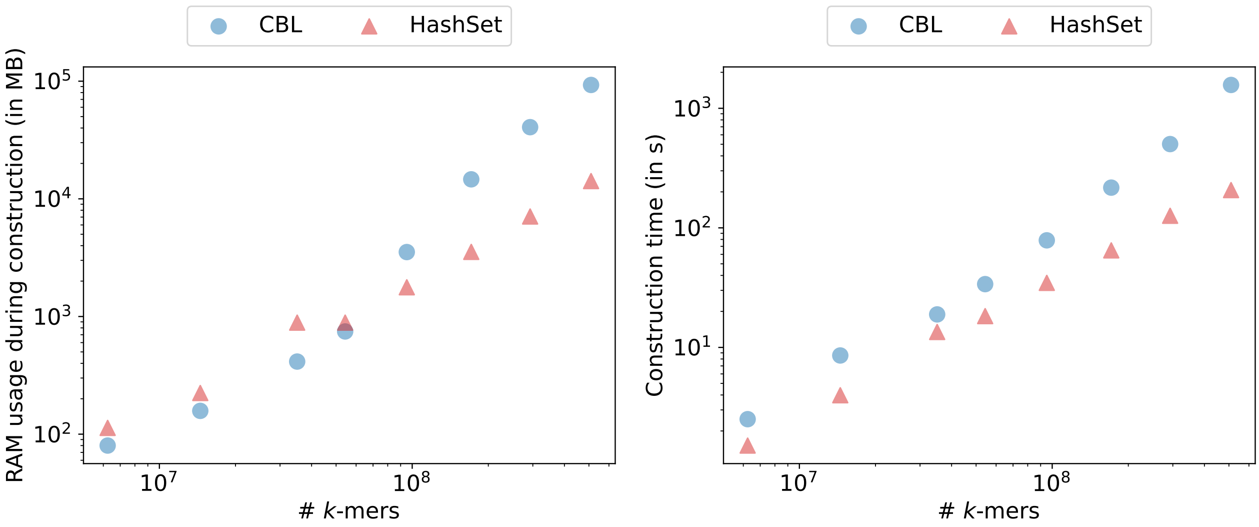

Figure S1: Time and RAM used during construction on indexes with growing number of k-mers from bacterial genomes, for a prefix size of 22.

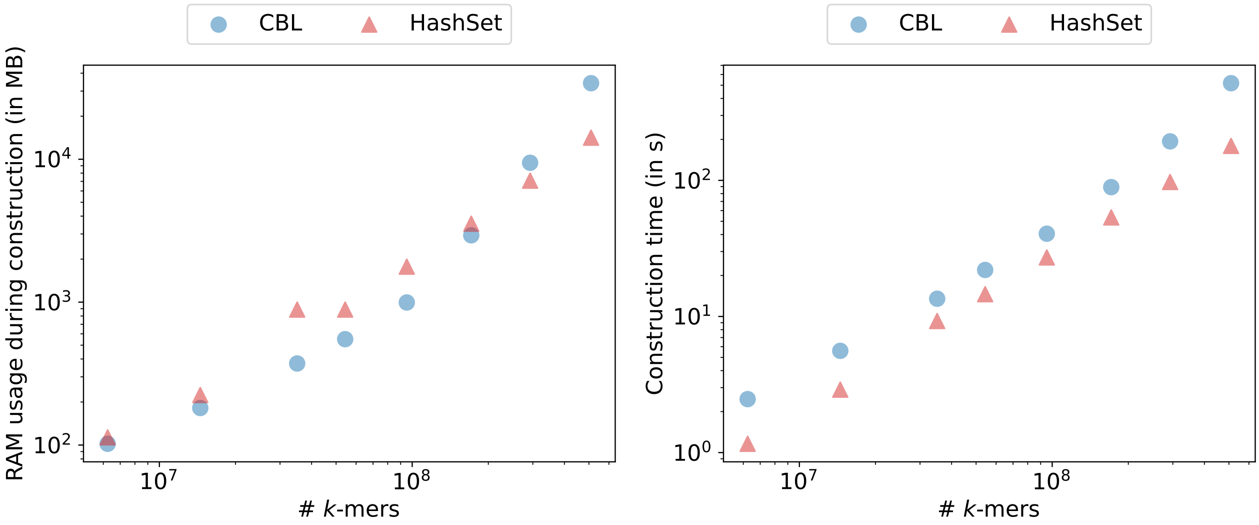

Figure S2: Time and RAM used during construction on indexes with growing number of k-mers from bacterial genomes, for a prefix size of 24.

Differences in RAM usage between sizes 22 and 28 can be explained by the fact that increasing the prefix size on large instances implies that tries of suffixes are less populated, which decrease their overhead. Conversely, jumps to other suffixes buckets become more frequent during accesses.

Benchmarks on human RNA-seq

Illumina raw reads FASTA files were downloaded from SRA with accessions SRR972708, SRR975415, SRR962597, SRR976738, SRR953494, SRR950080, SRR950083, SRR953488, SRR972717, SRR972716, SRR975416, SRR975412, SRR962604, SRR976749, SRR953495. Unitigs were built using BCALM2, keeping k-mers with a multiplicy greater than 2. Additional files were used for query experiments, obtained in a similar setting: SRR975414, SRR950879, SRR976743, SRR950882, SRR950881, SRR950079, SRR972713, SRR972715, SRR960732, SRR960733, SRR975411, SRR962602, SRR962600, SRR950084.

We display here similar analysis of the main document, benchmarking index building, queries, insertions and deletions.

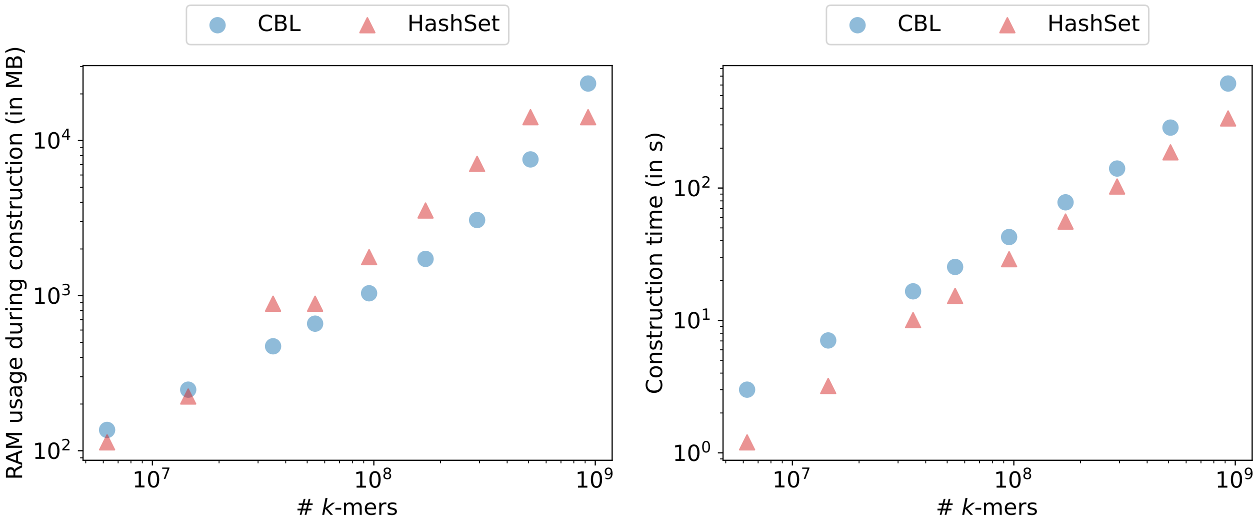

Figure S3: Time and RAM used during construction on indexes with growing number of k-mers from bacterial genomes, for a prefix size of 26.

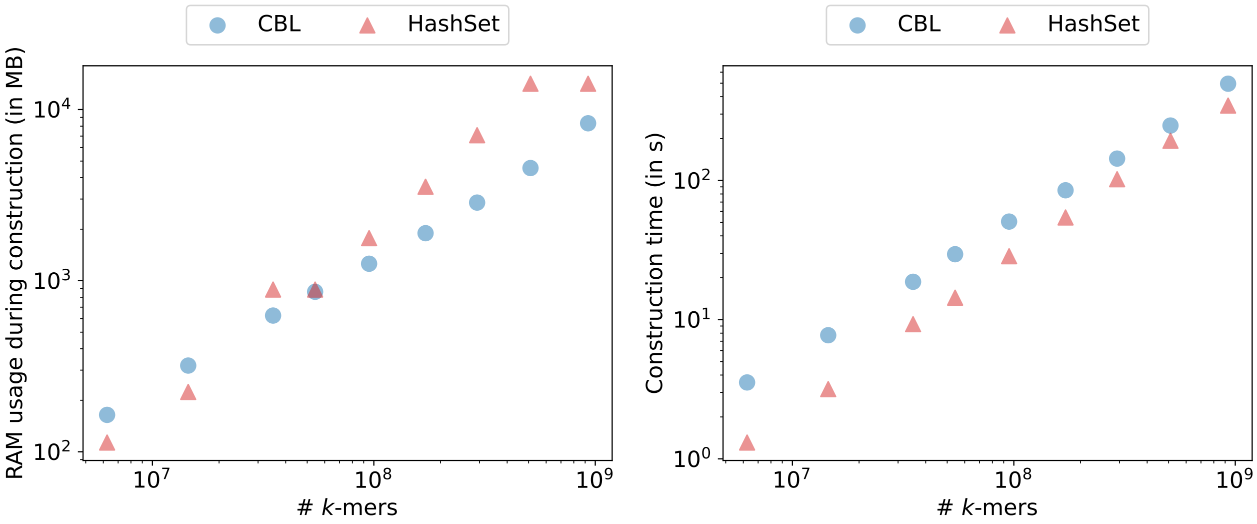

Figure S4: Time and RAM used during construction on indexes with growing number of k-mers from bacterial genomes, for a prefix size of 28.

Benchmarks on HiFi reads

We report a building benchmark on a growing amount of HiFi reads from a *E.coli* sequencing (accession SRR11434954)

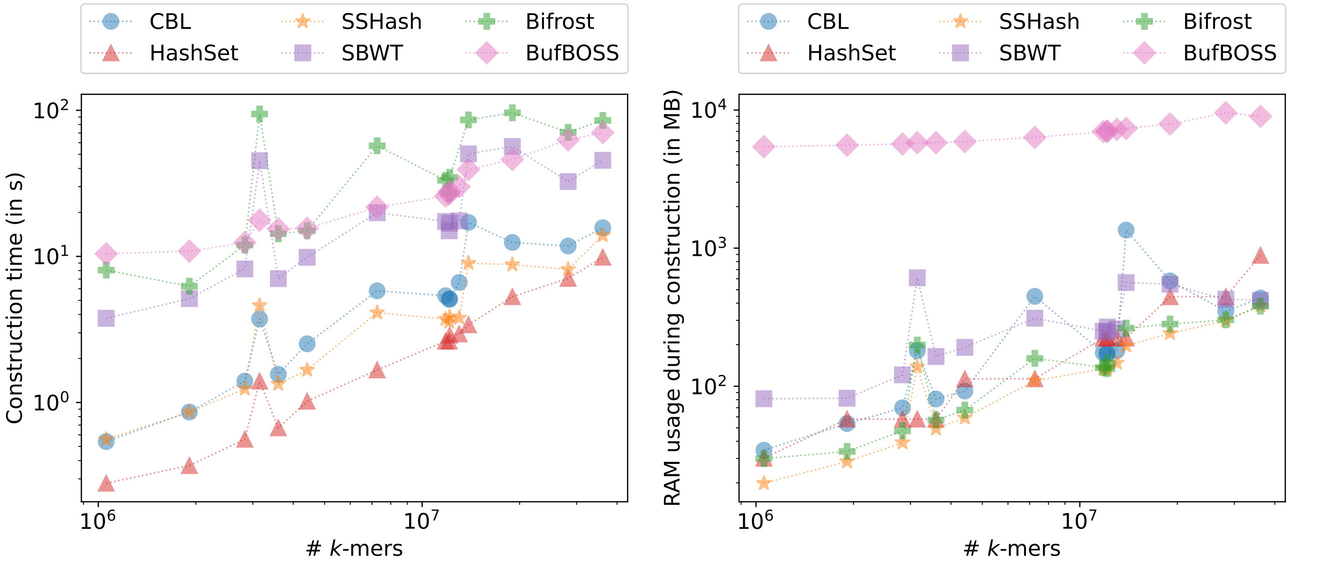

Figure S5: Time and RAM used when construction various indexes on growing number of k-mers from unitigs built on human RNA-seq.

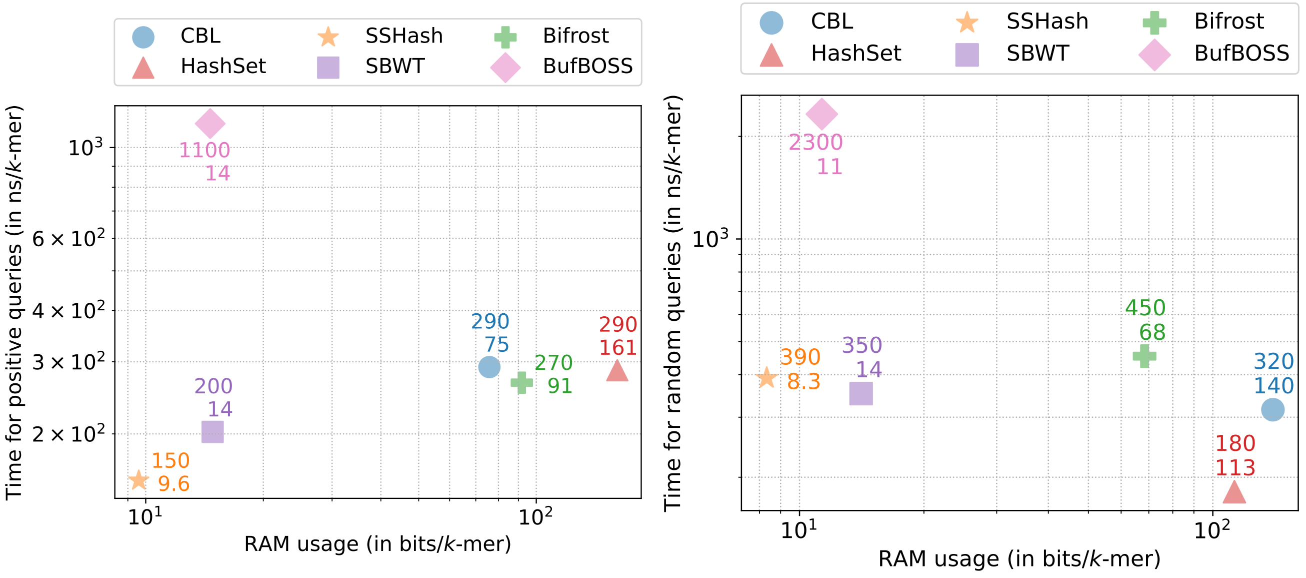

Figure S6: Time/memory trade-off of various tools when performing positive queries (up) and negative queries (down)

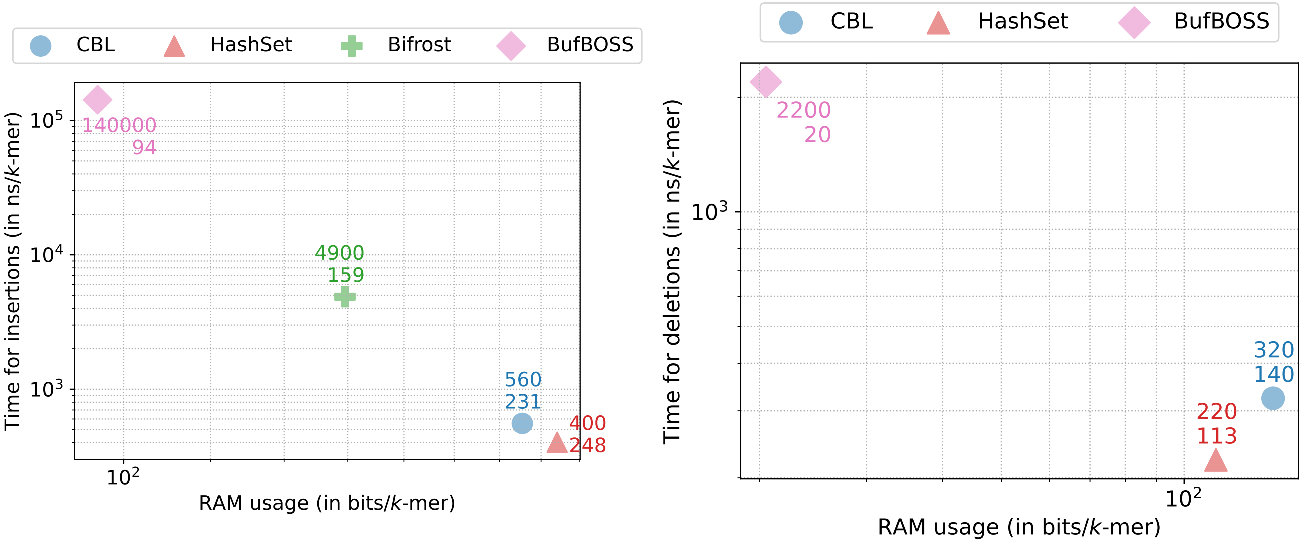

Figure S7: Time/memory trade-off of various tools when performing positive queries (up) and negative queries (down)

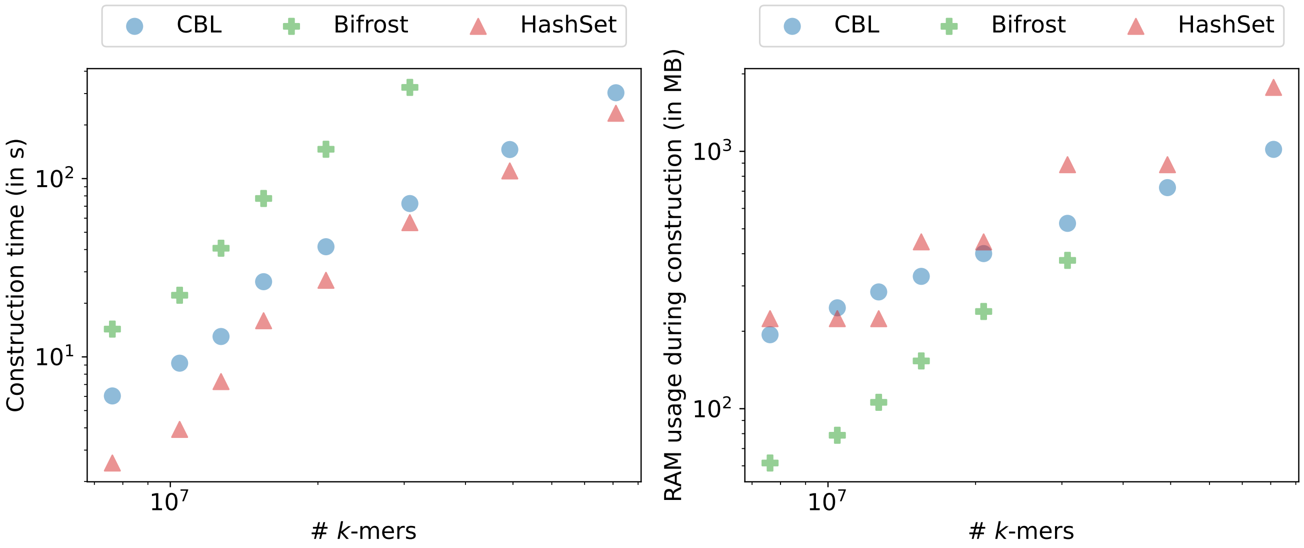

Figure S8: Time and RAM used when constructing various indexes on a HiFi long read dataset for $k = 31$ and $p = 28$ bits.
